# Effects of endurance exercise training on endoplasmic reticulum stress in pancreatic islets of obese mice

**DOI:** 10.1590/1414-431X2025e14499

**Published:** 2025-06-20

**Authors:** E. Marconato-Júnior, G.M. Soares, K. Rodrigues-dos-Santos, T. dos Reis Araujo, J.F. Vettorazzi, L. Zangerolamo, J.M. Costa-Junior, E.M. Carneiro, A.C. Boschero, H.C.L. Barbosa

**Affiliations:** 1 Centro de Pesquisa em Obesidade e Comorbidades, Departamento de Biologia Estrutural e Funcional, Instituto de Biologia, Universidade de Estadual de Campinas, Campinas, SP, Brasil; 2 Instituto Latino-Americano de Ciências da Vida e da Natureza, Universidade Federal da Integração Latino-Americana, Foz Do Iguaçú, PR, Brasil.

**Keywords:** Endurance exercise, Pancreatic islet, Obesity, Type 2 diabetes mellitus, Glucose homeostasis, Endoplasmic reticulum stress

## Abstract

Obesity is a serious health problem worldwide and the search for new control methods and therapies is imperative. Studies indicate that a variety of obesogenic diets may increase the risk of developing type 2 diabetes mellitus (T2D) by causing insulin resistance in peripheral tissues. The chronic increase in free fatty acids associated with obesity may increase insulin demand by pancreatic beta cells and induce intrinsic beta cell dysfunction through endoplasmic reticulum (ER) stress, which is associated with beta cell loss during the development of T2D. Physical exercise approaches have been emerging as powerful tools and adjuncts in a variety of conditions, improving glucose homeostasis, oxygen uptake, and metabolism. Here, we showed that a 16-week endurance training program mitigated the deleterious effects of an obesogenic diet on glycemic homeostasis, insulin secretion, and ER stress markers as well as islet health markers in C57/BL6 obese mice. The results corroborated the assumption that physical exercise is an effective therapy to avoid beta cell death in glucose metabolism dysfunction and T2D in obese individuals.

## Introduction

Obesity is becoming a serious condition worldwide, requiring new methods and effective therapies (1). The worsening of obesity leads to insulin resistance, increasing the demand on pancreatic beta cells to produce and secrete insulin. Hypersecretion of insulin worsens the obese state and, over time, it may lead to failure of beta cells to maintain hypersecretion triggering type 2 diabetes mellitus (T2D) (2-4).

Studies indicate that a high-fat diet (HFD) may increase the risk of developing T2D. The chronic increase in free fatty acids associated with obesity may increase insulin demand by pancreatic beta cells and induce intrinsic beta cell dysfunction through endoplasmic reticulum (ER) stress (5,6). ER stress is a mechanism related to the progressive decline in beta cell function and mass during progression to T2D (4,7,8).

Physical exercise is known to be a powerful tool and adjunct in the treatment of a variety of conditions (9-12) by improving glucose homeostasis, oxygen uptake, and metabolism (13-15).

The search for treatment, new therapies, and light-to-moderate impact exercise protocols for glycemic control are essential in a scenario where many people are affected by obese comorbidities like diabetes. Endurance exercise is an important strategy to improve glycemic control and beta cell function, as previously reported (16-19).

Part of the beneficial effects of physical exercise is through molecules known as exerkines, and some reports have shown their contribution to beta cell features, such as survival and proliferation. However, there is little information on the mechanisms related to the direct beneficial effects of exercises and exerkines on beta cells or pancreatic islet function (reviewed in (20)). Previous studies show that exposure of pancreatic beta cells to a diabetes environment and treated with serum from endurance trained animals improves viability and function of these cells (21). Indeed, the downregulation of ER stress-associated genes has been observed in the islets of resistance-trained mice. In addition, the serum of exercise-trained mice was able to reduce the dysfunction caused by cyclopiazonic acid (CPA), an ER stress inducer, on the cell function of the rat insulinoma cell line (INS-1E), indicating that exerkine may play a role in ER stress-associated dysfunction in beta cells (22). Interleukin 6 (IL-6), a molecule stimulated by physical exercise, was shown to enhance glucose-stimulated insulin secretion, possibly via a phospholipase C (PLC)-inositol trisphosphate (IP3)-dependent pathway (23). Another skeletal muscle cytokine, C-X3-C motif chemokine ligand 1 (CX3CL1), was reported to be involved in glucose-stimulated beta cell insulin secretion in a CX3CR1- and mitogen-activated protein kinase (MEK)-dependent way, inducing insulin secretion and maintaining beta cell function (24).

Irisin, an adipomyosin released during exercise, was also reported to promote beta cell survival, glucose-stimulated insulin secretion, and induced beta cell proliferation (25,26). Another study showed that irisin was capable of promoting beta cell proliferation, possibly via the ERK-mitogen-activated protein kinase (MAPK) pathway and inhibition of beta cell apoptosis through regulation of caspase levels (27).

Moreover, the interaction between the nervous system and beta cells during exercise is a rising topic for the emerging neuroendocrinology of islet function and will certainly bring novel insights into the molecular mechanisms of therapeutic exercise intervention (20).

In the present study, we aimed to evaluate the effect of endurance training on the deleterious effects of obesity including glycemic homeostasis, insulin secretion, and ER stress markers and islet health markers in C57/BL6 mice fed a high-fat diet (HFD).

## Material and Methods

### Animals

All animal experiments were carried out in accordance with the protocols approved by the Animal Care and Use Committee of the University of Campinas (UNICAMP) (number: 5023-1/2018) and the last revision of the National Institutes of Health (NIH) Guide for the Care and Use of Laboratory Animals. The study was carried out in compliance with the ARRIVE guidelines (https://arriveguidelines.org). C57BL/6 mice were obtained from UNICAMP and maintained at 23±1°C in a 12-h light/dark cycle.

### High-fat diet

Ninety-day-old mice were assigned to the following groups: control (Ctl), endurance trained (Ex), high-fat diet-fed (HFD), and high-fat diet-fed endurance-trained (HFD-Ex). The treatment was performed for 16 weeks. Groups Ctl and Ex were fed a diet with normal fat content (Nuvilab, Quimtia, Brazil), while HFD and HFD-Ex animals were fed a high-fat diet (60%) (PragSoluções, Brazil). Diet compositions are described in Table 1. The body weight of all mice was evaluated for 16 weeks, during all treatments.

**Table 1:** Composition of the diets.

Ingredients (g/kg)	Control diet	High-fat diet
Casein	140	140
Cornstarch	465.7	208.7
Dextrinized cornstarch	155	100
Sucrose	100	100
L-cystine	1.8	1.8
Fiber	50	50
Soybean oil	40	40
Mineral mix (AIN-93M)	35	35
Vitamin mix (AIN-93M)	10	10
Choline chlorhydrate	2.5	2.5
Lard	-	312
Energy (kcal/kg)	3.88	5.44

### Endurance training

For training, the animals were acclimated to the treadmill in individual stalls and no shock. The protocol lasted 16 weeks, in which the animals ran at a speed corresponding to 70% of their maximum limit capacity. The sessions started with 10 min duration and gradually increased to 60 min a day, 5 days a week. All training sessions for each group occurred at 2:00 p.m. To assess the maximum effort capacity, the running test was performed on the treadmill, in which the animals started running at a speed of 5 cm/s and increased by 3 cm every minute until the animal went into exhaustion. The test was carried out before, in the middle of, and at the end of the training program to evaluate the performance of the animals and adjust the percentage of speed during the training program.

### Body parameters and blood sample collection

The mice were euthanized by decapitation (for blood collection), after isoflurane inhalation. Blood samples were collected and centrifuged at 950 *g*, 4°C for 15 min to obtain serum, which was stored at -20°C until insulin levels were subsequently measured and quantified using Rat/Mouse Insulin ELISA kit (Merck Millipore, Germany). After an abdominal incision, the inguinal, retroperitoneal, and perigonadal fat pads and the gastrocnemius muscle were dissected and weighed.

### Intraperitoneal glucose tolerance test

To perform the intraperitoneal glucose tolerance test (ipGTT), the animals were fasted for 12 h, and fasting glucose was evaluated using the Accu-Check Advantage II device (Roche, Switzerland). Then, glucose (1 g/kg body weight) was administered intraperitoneally and the glycemia values were measured 15, 30, 60, 90, and 120 min after the glucose challenge.

### Intraperitoneal insulin tolerance test

To assess insulin sensitivity, an intraperitoneal insulin tolerance test (ipITT) was performed five days after the ipGTT. The animals were fasted for 4 h and the glycemia was checked using the Accu-Check Advantage II device (time 0). Subsequently, regular human insulin was administered intraperitoneally (1 U/kg body weight). Glycemia was verified at 3, 6, 9, 12, 15, and 18 min. The constant rate for glucose disappearance (kITT) was calculated as previously described (28).

### Homeostasis model assessment of insulin resistance

Homeostasis model assessment of insulin resistance (HOMA-IR) was calculated according to the following formula: HOMA-IR = (fasting glucose (mmol/L) × fasting insulin (mU/L) / 22.5) (29).

### Glucose-stimulated insulin secretion in pancreatic islets

After an abdominal incision, 2.5 mL Hanks' solution enriched with 0.8 mg/mL collagenase (Sigma-Aldrich Chemical Co., USA) was injected into the pancreas through the common bile duct, and the pancreas was then dissected and transferred to a 50 mL tube and kept in a bath at 37°C for 17 min. Groups of four islets were pre-incubated for 30 min in Krebs-Ringer bicarbonate (KRB) buffer (115 mM NaCl, 5 mM KCl, 10 mM NaHCO_3_, 2.56 mM CaCl_2_, 1 mM MgCl_2_, and 15 mM HEPES; Sigma-Aldrich Chemical Co.), supplemented with 5.6 mM glucose plus 0.3% of BSA, and equilibrated with a mixture of 95% O_2_/5% CO_2_ to give pH 7.4 for 45 min at 37°C. Subsequently, the islets were exposed to 1 mL KRB containing 2.8 or 16.7 mM glucose for 1 h at 37°C. To measure secreted insulin, we collected the supernatants that were stored at -20°C, and the remaining islets were homogenized in an alcohol/acid solution to measure the total insulin content. Rat/Mouse Insulin ELISA kit was used to measure and quantify insulin levels (Merck Millipore). Insulin secretion was normalized by total insulin content.

### Quantitative PCR

Groups of 200 islets were collected, and RNA was extracted using TRIzol reagent (Life Technologies, USA) to obtain the total RNA content of the isolated islets following phenol-chloroform RNA extraction, according to the manufacturer's recommendations. RNA concentration was measured by SpectraMax^®^ i3x Multi-Mode Microplate Reader (Molecular Devices, LLC, USA). cDNA was prepared using 1 μg RNA and MultiScribe reverse transcriptase (Applied Biosystems, USA). The SYBR-green master mix (Applied Biosystems) was used in the PCR reactions. Quantification was performed using the 7500 Fast Real-time PCR System (Applied Biosystems). The specificities of amplifications were verified by melting-curve analyses. The relative expression of mRNAs was determined after normalization with *Hprt*, using the 2-ΔΔCT method. Primer sequences used for real-time PCR assays were as follows: *Pdx1* forward: 5′-gaacccgaggaaaacaagagg-3′, *Pdx1* reverse: 5′-gttcaacatcactgccagctc-3′; *Ins2* forward: 5′-ccagctaagacctcagggact-3′, *Ins2* reverse: 5′-ctggaagataggctgggttgag-3; *Atf4* forward: 5′-gttggtcagtgcctcagaca-3′, *Atf4* reverse: 5′-cattcgaaacagagcatcga-3; *Xbp1* forward: 5′-agcagcaagtggtggatttg-3′, *Xbp1* reverse: 5′-gagttttctcccgtaaaagctga-3; *Bip* forward: 5′-acttggggaccacctattcct-3′, *Bip* reverse: 5′-atcgccaatcagacgctcc-3′; *Chop* forward: 5′-ctggaagcctggtatgaggat-3′, *Chop* reverse: 5′-cagggtcaagagtagtgaaggt-3.

### Statistical analysis

The groups were analyzed by the two-way ANOVA test followed by the Bonferroni post-test (GraphPad Prism 5, USA). Data are reported as means±SEM, and the difference between the groups was considered statistically significant at P<0.05

## Results

### Endurance training prevented weight gain in obese mice

**Figure 1:**
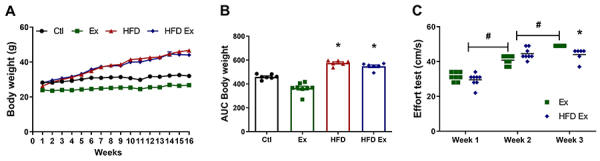
Endurance training prevented weight gain, and high-fat diet (HFD) impaired the physical capacity of mice. Body weight (**A**) and area under the curve (**B**) of control (Ctl), exercised (Ex), HFD, and high-fat diet exercised (HFD Ex) animals. **C**, Maximum effort test results of HFD and HFD-Ex animals. Data are reported as means±SEM (n=6-8). *P<0.05 compared to the standard diet control (Ctl) group; ^#^P≤0.05 in the indicated comparisons (two-way ANOVA followed by the Bonferroni post-test).

Throughout the 16-week study period, animals in the control group (Ctl) and trained group (Ex) showed stable body weight gain, as expected. Animals that received the HFD and animals that received the diet and were trained (HFD-Ex) had a significantly higher increase in weight compared to Ctl (Figure 1A), indicated by the greater area under the curve (AUC) (Figure 1B). Training was not able to prevent weight gain of the HFD animals over the 16 weeks, as shown in Figures 1A and B. In addition, the HFD animals that underwent endurance training had a lower maximum effort value on the treadmill test at week 16 of training compared to the Ex group (Figure 1C), indicating that HFD consumption impaired their performance on this test.

### Endurance training prevented inguinal and retroperitoneal fat pad deposition in obese mice

**Figure 2:**
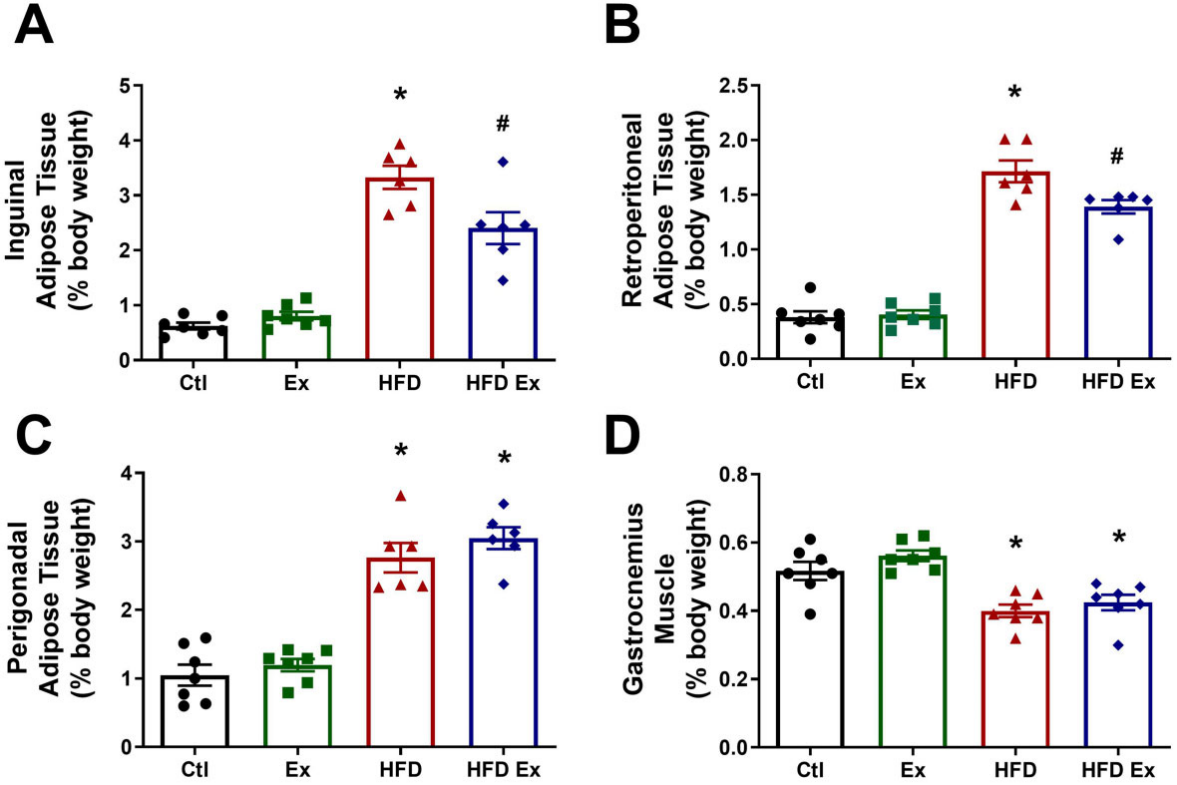
Effect of endurance training in inguinal and retroperitoneal fat pad deposition. Weight of inguinal (**A**), retroperitoneal (**B**), and perigonadal fat pads (**C**) and gastrocnemius muscle (**D**) of control (Ctl), exercised (Ex), high-fat diet (HFD), and high-fat diet and exercised (HFD-Ex) animals. Data are reported as means±SEM (n=6-7). *P≤0.05 compared to the Ctl group; ^#^P≤0.05 compared to the HFD group (two-way ANOVA followed by the Bonferroni post-test).

We observed a significant increase in the weight of inguinal, retroperitoneal, and perigonadal adipose tissue in the HFD group compared to Ctl. Animals in the HFD-Ex group, on the other hand, had lower inguinal and retroperitoneal adipose tissue weight (Figure 2A and B) compared to HFD animals at 16 weeks, but training did not prevent perigonadal adipose tissue weight gain (Figure 2C). In addition, the weight of the gastrocnemius skeletal muscle was lower in both the HFD and HFD-Ex groups, with no significant differences between them (Figure 2D). Thus, our results indicated that endurance training partially prevented fat deposition in inguinal and retroperitoneal adipose tissue.

### Endurance training improved glucose homeostasis in obese mice

**Figure 3:**
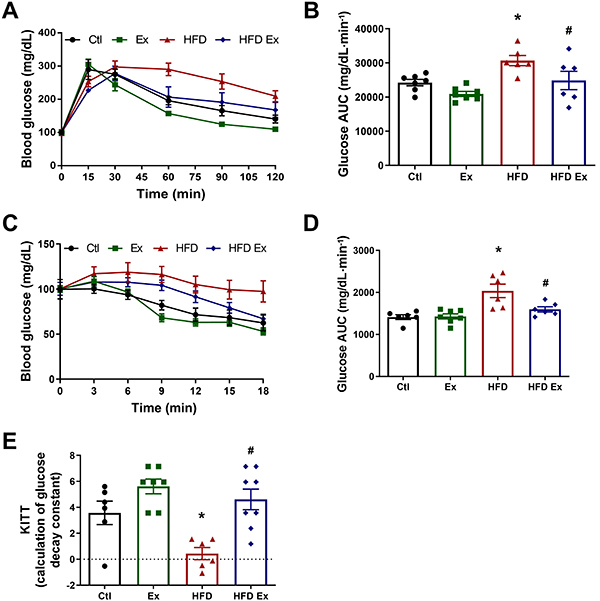
Effect of endurance training on glucose homeostasis and insulinemia. Blood glucose (**A**) and area under the curve (AUC) (**B**) in control (Ctl), exercised (Ex), high-fat diet (HFD), and high-fat diet exercised (HFD-Ex) mice after intraperitoneal glucose tolerance test. Blood glucose (**C**) and AUC (**D**) of Ctl, Ex, HFD, and HFD-Ex mice after the intraperitoneal insulin tolerance test and glucose disappearance rate (KITT) (**E**). Data are reported as means±SEM (n=6-8). *P≤0.05 compared to the Ctl group; ^#^P≤0.05 compared to the HFD group (two-way ANOVA followed by the Bonferroni post-test).

As expected, HFD-Ex animals were more tolerant to glucose than HFD animals in the ipGTT (Figure 3A and B). The HFD group had glucose intolerance compared to the Ctl group, indicated by the higher AUC (Figure 3B). To assess insulin resistance, the animals were fasted for 4 h and had their fasting blood glucose measured (time 0). As expected, HFD-Ex animals showed better insulin sensitivity than HFD animals. In the ipITT, the HFD group had insulin resistance compared to the Ctl group (Figure 3C), indicated by the higher AUC (Figure 3D). These data corroborated the glycemia decay constant (kITT) results, with the HFD group showing an impairment, while the HFD-Ex group presented a similar kITT to the Ctl group, as shown in Figure 3E. The data showed that endurance training improved glucose and insulin tolerance in mice fed a HFD for 16 weeks.

### Endurance training prevented hyperglycemia and hyperinsulinemia and improved insulin secretion in obese mice

**Figure 4:**
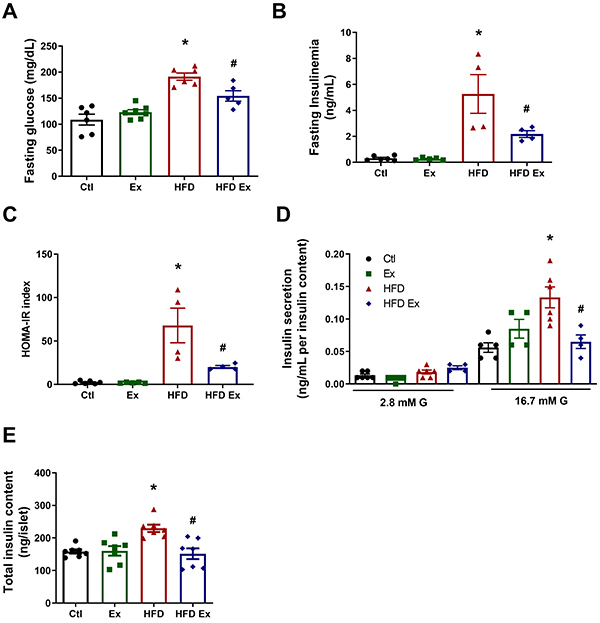
Effect of endurance training on hyperglycemia, hyperinsulinemia, and insulin secretion in mice. Fasting glycemia (**A**) and insulinemia (**B**) of control (Ctl), exercised (Ex), high-fat diet (HFD), and high-fat diet exercised (HFD-Ex) mice. Homeostatic model assessment of insulin resistance (HOMA-IR) (**C**) of Ctl, Ex, HFD, and HFD-Ex mice. Insulin secretion of isolated pancreatic islets after 1 h incubation with 2.8 and 16.7 mM glucose (**D**), and total insulin content (**E**) of Ctl, Ex, HFD, and HFD-Ex mice. Data are reported as means±SEM (n=4-7). *P<0.05 compared to the Ctl group; ^#^P≤0.05 compared to the HFD group (two-way ANOVA followed by the Bonferroni post-test).

In the fasting glycemia test, the HFD group presented higher glycemia than the Ctl group (Figure 4A), while the HFD-Ex group presented lower glycemia compared to the HFD group. In addition, we evaluated the fasting insulinemia of the groups (Figure 4B), which corroborated the fasting blood glucose values, evidenced by the expected high levels in the HFD mice and lower insulinemia in the HFD-Ex group. The HOMA-IR index was also reduced in HFD-Ex mice (Figure 4C), reinforcing the benefits of exercise in improving insulin resistance. Induced insulin secretion and total insulin content in isolated pancreatic islets were higher in the HFD group compared to the Ctl group, while levels in the HFD-Ex group were similar to the Ctl group (Figure 4D and E). These data demonstrated the beneficial effects of endurance exercise both on glucose and insulin homeostasis in obese trained mice. The effects of physical exercise can also improve insulin sensitivity in peripheral tissues. Therefore, the lower insulin levels observed in the HFD-Ex group may reflect better insulin signaling and consequently improved glucose homeostasis.

### Endurance training ameliorated ER stress in isolated pancreatic islets in obese mice

**Figure 5:**
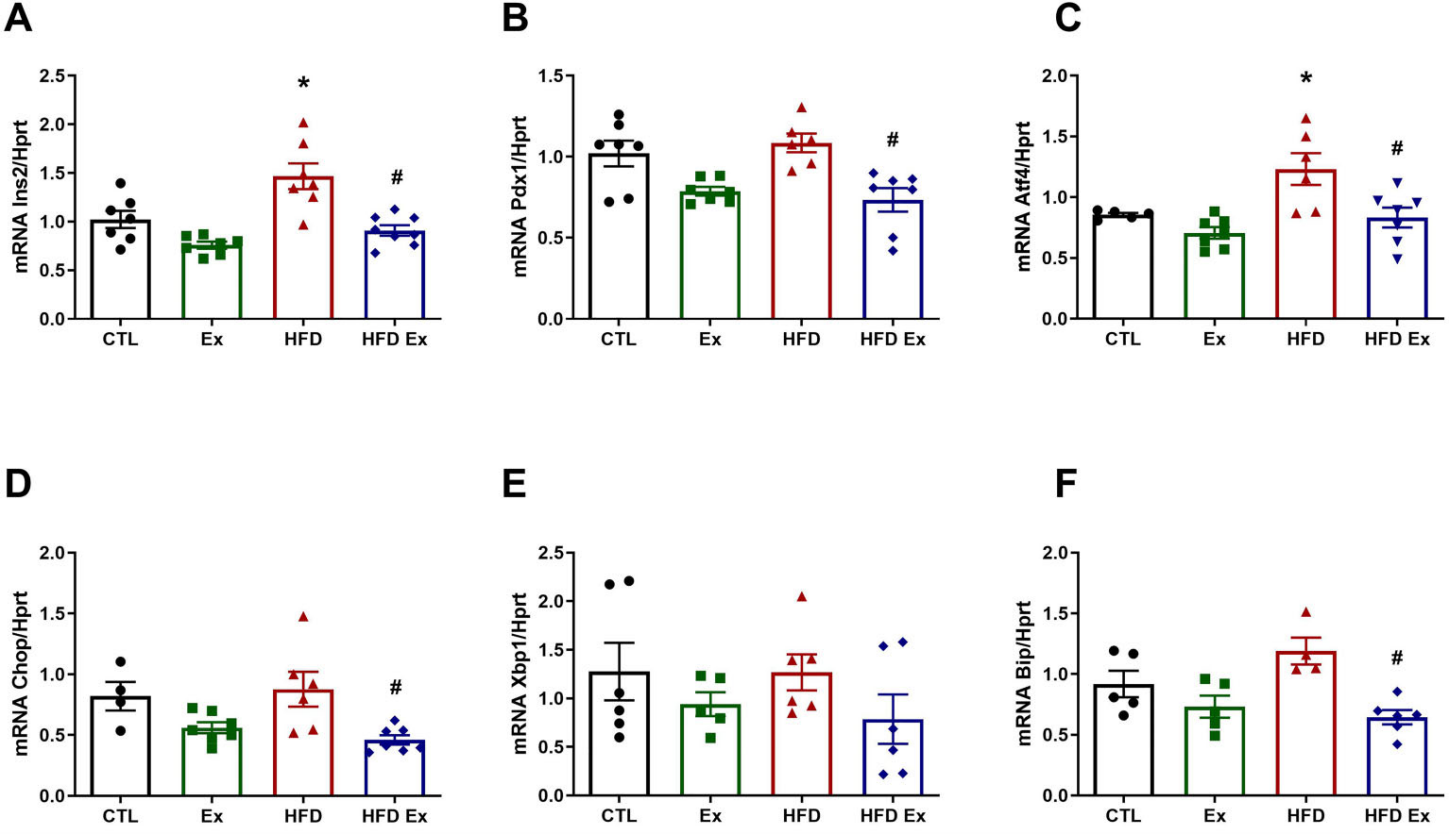
Gene expression of *Ins2* (**A**), *Pdx-1* (**B**), *Atf4* (**C**), *Chop* (**D**), *Xbp1* (**E**), and *Bip* (**F**) in isolated pancreatic islets of control (Ctl), exercised (Ex), high-fat diet (HFD), and high-fat diet exercised (HFD-Ex) mice. The samples were normalized by the *Hprt* housekeeping gene. Data are reported as means±SEM (n=4-8). *P≤0.05 compared to the Ctl group; ^#^P≤0.05 compared to the HFD group (two-way ANOVA followed by the Bonferroni post-test).

As HFD treatments imply different deficiencies in the islets and beta cell function, we assessed important markers of beta cell function, the regulation of multiple genes involved in ER homeostasis and their unfolded protein response (UPR), like insulin (*Ins2*), pancreatic and duodenal homeobox 1 (*Pdx1*), and ER stress markers in isolated pancreatic islets by quantitative PCR. Due to insulin secretion and content results in the experimental groups, mRNA expression analysis was performed in isolated pancreatic islets. As already established in the literature, *Ins2* gene expression was higher in the HFD group compared to the Ctl group and lower in the HFD-Ex group, compared with the HFD group (Figure 5A). These results are in agreement with the hormone secretion assay results in isolated islets and with the plasma insulin concentration in the experimental groups (Figure 4B and C). Furthermore, *Pdx-1* gene expression was lower in the HFD-Ex compared to the HFD group, with no significant difference from the Ctl group (Figure 5B). Expression of activating transcription factor 4 (*Atf4*), an important marker of ER stress and UPR activation, was increased in the HFD group compared to the Ctl group (Figure 5C). No significant differences were found in CCAAT-enhancer-binding protein homologous protein (*Chop*), transcription factor x-box binding protein (*Xbp1*), and binding immunoglobulin protein (*Bip*) markers (Figure 5D-F) between the HFD and Ctl groups. Physical training prevented the increase in *Atf4*, *Chop*, and *Bip* gene expressions, as observed in the HFD-Ex group compared to the HFD group (Figure 5C-F).

## Discussion

In the present study, we found that physical training can decrease and mitigate the effects of HFD on glycemic homeostasis, insulin secretion, and ER stress markers and islet health markers in C57/BL6 obese mice.

All the evaluated parameters, such as body weight and adiposity, indicated that the HFD induced deleterious effects by nutritional excess. Animals fed a HFD and submitted to physical exercise had lower insulinemia and blood glucose and normal insulin tolerance, which indicated that physical exercise modulated glycemic homeostasis and insulin sensitivity and secretion. In addition, physical exercise protected the glycemic homeostasis from the deleterious effects of HFD. Glucose-induced insulin secretion by isolated islets was higher in the animals fed the HFD compared to the Ctl group, as expected, and lower in exercised HFD animals compared to HFD mice. It is known that exercise can improve insulin sensitivity due to changes in the expression and/or activity of proteins involved in insulin signal transduction in skeletal muscle (30) and liver (31) in rodents. Moreover, endurance exercise was able to maintain a normal pancreatic beta cell insulin secretion (16,21). Calegari et al. (16) showed that endurance exercise improved anti-apoptotic protein levels, while pro-apoptotic proteins were significantly suppressed in healthy rats, which could be associated with increased levels of pERK.

Our data showed that endurance exercise protected pancreatic islets by decreasing the expression of UPR genes such as *Atf4*, *Chop*, and *Bip* in obese mice, corroborating the few studies that evaluated beta cells or pancreatic islets *in vitro* (17,19). We observed that endurance training prevented an increase in the expression of canonical ER stress markers in obese animals, even when the diet was unable to induce increased expression of these genes in the HFD group, as previously reported (32). Another *in vivo* evidence of exercise effects was previously reported by our group, showing that resistance training promoted downregulation of ER stress-associated genes in the islets of mice (22). Although the HFD did not induce an increase in some UPR markers in pancreatic islets, all other parameters evaluated, such as body weight, adiposity, hyperinsulinemia, glucose intolerance, and insulin resistance, indicated that the diet induced the deleterious effects expected from nutritional overload. These effects were mitigated by the 16-week endurance training. In animals fed a HFD and submitted to physical exercise, there was a decrease in the expression of ER stress markers compared to the HFD group, indicating the protective effect of exercise on UPR markers gene expression. In our study, the increase in *Atf4* gene expression induced by HFD stands out, indicating a possible moderate metabolic stress in pancreatic islets, since nutritional overload led to an UPR marker activation. Thus, we could infer that the training program attenuated the activation of one of the pathways associated with ER stress. Also, a known ER homeostasis regulator, *Pdx1*, was also reduced by the training program, as was *ATF4* (33).

Another important observation is that many exerkines are released during physical training by skeletal muscle tissue (16,17,19) that could be involved in the *in vivo* effects observed in our study. There is evidence that these molecules could modulate many aspects of pancreatic beta cells, but most of the experiments were performed in isolated islets *in vitro* or lineage beta cells (23,). Thus, it is possible that myokines released in plasma in our endurance training model could help prevent ER stress in obese mice, as observed in our study. More studies are necessary to elucidate the effects of endurance training on pancreatic islets and their contributions to the prevention of obesity-associated dysfunctions. Our data corroborated the assumption that physical exercise, even without great weight loss, is capable of improving beta cell function and glucose/insulin homeostasis, and could be beneficial as a complementary treatment to prevent insulin resistance or T2D progression.

In conclusion, our work showed through *in vivo* evidence that exercise can modify the response of pancreatic islets to a HFD in obese mice. Further studies are needed to better understand the mechanisms involved in the compensatory response in obese individuals in order to avoid the development and progression of T2D.
